# Validation of a Hyperspectral Imaging System for Color Measurement of In-Vivo Dental Structures

**DOI:** 10.3390/mi13111929

**Published:** 2022-11-09

**Authors:** Maria Tejada-Casado, Razvan Ghinea, Miguel Ángel Martínez-Domingo, María M. Pérez, Juan C. Cardona, Javier Ruiz-López, Luis Javier Herrera

**Affiliations:** 1 Department of Optics, Faculty of Science, Campus Fuentenueva, Edificio Mecenas, s/n., University of Granada, ibsGranada, 18071 Granada, Spain; 2 Instituto de Investigación Biosanitaria ibs.GRANADA, 18012 Granada, Spain; 3 Computer Architecture and Technology Department, University of Granada, 18071 Granada, Spain

**Keywords:** hyperspectral imaging, color, dentistry

## Abstract

A full comprehension of colorimetric relationships within and between teeth is key for aesthetic success of a dental restoration. In this sense, hyperspectral imaging can provide point-wise reliable measurements of the tooth surface, which can serve for this purpose. The aim of this study was to use a hyperspectral imaging system for the colorimetric characterization of 4 in-vivo maxillary anterior teeth and to cross-check the results with similar studies carried out with other measuring systems in order to validate the proposed capturing protocol. Hyperspectral reflectance images (Specim IQ), of the upper central (UCI) and lateral incisors (ULI), were captured on 30 participants. CIE-L*a*b* values were calculated for the incisal (I), middle (M) and cervical (C) third of each target tooth. $\Delta E_{ab}^{*}$ and $\Delta E_{00}$ total color differences were computed between different tooth areas and adjacent teeth, and evaluated according to the perceptibility (PT) and acceptability (AT) thresholds for dentistry. Non-perceptible color differences were found between UCIs and ULIs. Mean color differences between UCI and ULI exceeded AT ($\Delta E_{ab}^{*}$ = 7.39–7.42; $\Delta E_{00}$ = 5.71–5.74) in all cases. Large chromatic variations between I, M and C areas of the same tooth were registered ($\Delta E_{ab}^{*}$ = 5.01–6.07 and $\Delta E_{00}$ = 4.07–5.03; $\Delta E_{ab}^{*}$ = 5.80–8.16 and $\Delta E_{00}$ = 4.37–5.15; and $\Delta E_{ab}^{*}$ = 5.42–5.92 and $\Delta E_{00}$ = 3.87–4.16 between C and M, C and I and M and I, respectively). The use of a hyperspectral camera has proven to be a reliable and effective method for color evaluation of in-vivo natural teeth.

## 1. Introduction

Human teeth consist of soft tissue (the pulp) covered by two hard tissues: dentin and enamel. Both of these tissues are very complex and have very characteristic optical properties. Tooth color is mainly determined by dentine, with the enamel playing a minor role, due to its translucency [1,2,3]. However, it has to be considered that the final color of a tooth is also affected by the thickness of the dentine and enamel layers, which has an influence on the light transmission, as it was previously reported [4,5,6]. Moreover, since the thickness of the layers is not constant along the entire tooth structure, but it varies according to the different tooth areas [7], it creates a color variation (gradient) along the different axis of the natural dental structures. These color variations within dental structures [8,9,10,11] are very difficult to measure with conventional devices used in clinical scenarios, which usually determine color by single spot measurements or large integrating measuring areas.

Colorimeters, spectrophotometers and digital imaging systems are, currently, among the most popular instruments for objective color measurements in dentistry [10]. All these instruments have their pros and cons. Colorimeters show good measurement repeatability but they are exposed to systematic errors caused by edge-loss effects from sample surface [12]. Spectrophotometers can provide more systematic and precise measurements than colorimeters or digital imaging methods [11,13,14,15] due to their ability to measure the amount of light reflected throughout the visible spectral range, although they require calibration for proper measurement [11]. Lastly, digital imaging systems represent the most basic approach for color measurement in dentistry, and their use is increasingly popular in the dental community due to the possibilities they provide in combination with editing tools and additional software [16,17]. However, they lack of systematization, require calibration and a certain degree of subjective shade selection involving the human eye [18].

On the other hand, the recent incorporation of spectroradiometers for color measurements in dental research provide accurate and highly repeatable non-contact measurements. Spectroradiometers are recognized as the gold standard devices for objective accurate color measurements and have been widely used in many research studies for the evaluation of the colorimetric and optical properties of dental materials [19,20,21,22,23,24]. One of the main advantages of these devices is that they measure complete spectral data, which provide information on the consistency of the material and can detect potential problems of metamerism, allowing to calculate colorimetric data for the actual spectral power distribution of each source expected to illuminate the specimen. However, their main constraint in the dental field is the impracticality of their use in clinical scenarios. This adds up to other limitations, shared with other clinical commercial devices, such as single spot or large integrated area of measurement. So far in dentistry, spectroradiometric color measurement is used exclusively for research purposes [22].

In this sense, hyperspectral imaging systems [25,26] have the potential to solve the majority of the problems previously described. Hyperspectral cameras increase the number of channels -from 3 in conventional imaging systems to tens or hundreds-, by imaging narrow contiguous wavelength bands, which means that they can provide pixel-wise reliable spectral reflectance measurements of the complete tooth surface. In dentistry, hyperspectral imaging systems have been used to acquire an extensive database of spectral images for their use in oral dental diagnostics [27]. They acquired a total of 316 oral and dental spectral reflectance images, which provides a vast amount of data that can be used for development, pattern recognition and machine vision applications for dentistry. However, to the best of our knowledge, these spectral images or similar techniques have not yet been used to evaluate color, or color variations, within and between dental structures.

When referring to tooth color, the typical reference is made to the color of its middle third (specially for frontal teeth). Although this approach has proven to be useful to date, one cannot overlook the fact that it is a somewhat limited approximation that may not be representative for the chromatic appearance of the tooth as a whole. Different studies [9,28] have attempted to determine the color relation between these three tooth segments (cervical, middle and incisal) using different color measuring devices such as colorimeters and digital photography, while others [10,11], used a spectrophotometer for this purpose. All of them concluded that there are significant differences between different teeth and areas of the same tooth, and that, therefore, individual selection of shades for each tooth and different areas of a tooth should be considered in order to increase the success of the restorations. In this regard, being able to objectively and precisely characterize the complete color gradient of the tooth has the ability to greatly contribute to the accurate reproduction of the color of dental structures, which would be of great help both in clinical restorations as well as in the development of new prosthetic teeth by dental manufacturing companies.

Taking into account all the above mentioned, the aim of this work is to validate the use of a hyperspectral imaging device for in-vivo color measurement of the four maxillary anterior teeth by evaluating the correlations between contralateral and adjacent teeth and different areas of the same tooth, and cross-checking the obtained results with those available in current literature. The research hypotheses tested were (1) color differences between contralateral teeth do not exceed the perceptibility color difference threshold (PT) for dentistry while those between adjacent teeth do exceed the acceptability color difference threshold (AT) and (2) color differences between cervical, middle and incisal thirds exceed the AT for dentistry.

## 2. Materials and Methods

### 2.1. Participants

A group of 30 volunteers (50% men and 50% women, aged between 21 and 67 years—average age of 29 years) were selected to participate in our research. Prior to be enrolled in the study, all participants received Information Sheets and signed an Informed Consent. The experimental protocol followed the guidelines of the Code of Ethics of the World Medical Association (Declaration of Helsinki 2013) and was approved by the University Ethics Committee (REF: 2032/CEIH/2021).

Before starting hyperspectral image capture, all participants were screened to evaluate the target teeth—maxillary anterior upper lateral incisors (right ULI1; left ULI2) and upper central incisors (right UCI1 and left UCI2)—to ensure the viability of the measurements. Presence of color alterations, restorations, remains of orthodontic cement, fractures, orthodontic retainers, alterations in position or any circumstance that alters or prevents the measurement of the color of the target teeth, were considered as exclusion criteria for participation.

### 2.2. Experimental Set-Up

**Figure 1:**
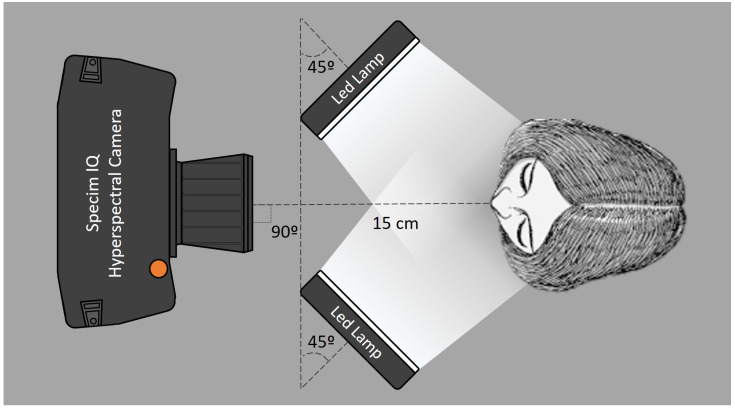
Schematic representation of the measurement set-up used to measure the hyperspectral reflectance images of the four maxillary incisors.

The experimental device was composed of a hyperspectral camera (Specim IQ, Spectral Imaging Ltd., Oulu, Finland), two LED lamps (ML46231122, BoliOptics, Rancho Cucamonga, CA, USA) and a chin rest. In order to maximize the size of the area being captured by the camera, this was placed 15 cm away from the target teeth, which is the minimum focus distance allowed by the system. Finally, the LED lamps were positioned so that a CIE $45^{\circ }/0^{\circ }$ illuminating/measuring geometry can be achieved (Figure 1).

Specim IQ is a portable hyperspectral camera with a wavelength range from 400 nm to 1000 nm. It has a spectral resolution of approximately 3 nm, resulting in a total of 204 spectral bands and a spatial resolution of 512 × 512 pixels. It is a line-scanning camera [26] with a constant integration time over the band images, therefore the whole spectrum is acquired at once and small involuntary movements do not affect individual bands of the spectrum. This camera also has an integrated RGB camera, which supports the spectral camera by allowing the visualization of the scene and adjusting the frame and focus in real time. The parallax between the two cameras can be adjusted so that the fields of view of both are the same.

### 2.3. Data Acquisition and Processing

Prior to the image acquisition, all participants brushed their teeth according to a standard brushing procedure (2 min brushing with a soft toothbrush and classic toothpaste), in order to remove any plaque or food debris. Then, participants were seated with their heads placed on a chin rest, in order to maintain the upper incisors always parallel to the objective lens of the hyperspectral camera. Each participant was provided with a sterilized lip retractor (906–2721, Henry Schein Inc., Melville, NY, USA) and was informed on how to keep their head still, placing the upper and lower incisors aligned and avoiding any contact of the tongue with the target teeth.

For every capture, the line integration time was set to 40 ms, being the image capturing time of roughly 8 s. This was proven to be high enough to provide good exposure without over-saturated pixel responses, and also without exceeding a whole capturing time (subject preparation, image focusing and image capturing) of 2 min, since in other studies was reported that, after this time, the degree of dehydration that occurs in the teeth modifies their color [29].

To correct the sensor spectral responses of the imaging system and the spectrally and spatially non-uniform illumination, the so-called “flat-field correction” [30], two extra black and gray images were captured using exactly the same settings employed to capture the target teeth. For the black image, the camera objective was covered while for the grey image a matt diffuse gray ceramic sample (Matt Diff Grey, CCSII, Lucideon, Staffordshire, UK) was used as suggested in [27], in order to avoid over-saturated responses.

**Figure 2:**
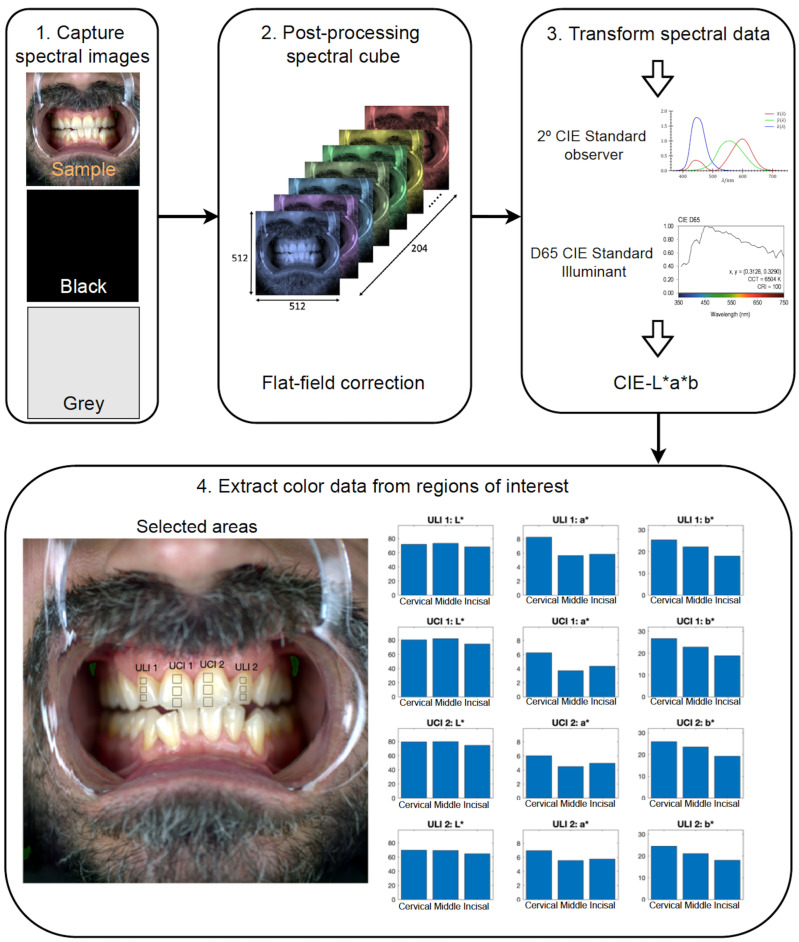
Workflow scheme of the spectral image capturing protocol, data processing and CIE L*, a* and b* values extraction corresponding to the cervical, middle and incisal thirds.

After correcting the spectral reflectance data, these were converted into CIELAB values using the CIE $2^{\circ }$ Standard Observer and the CIE D65 Standard Illuminant. Now, the chromatic variations along the tooth structures can be analyzed. For this purpose, each tooth was divided into three areas of interest known as the cervical third, middle third and incisal third, which correspond to the upper, middle and lower part of the tooth, respectively. From each of these three areas, the mean CIE L*, a* and b* values were extracted as shown in Figure 2.

### 2.4. Evaluation of Color Differences

Color differences between different target teeth and different tooth areas were computed using the CIELAB ($\Delta E_{ab}^{*}$) and CIEDE2000 ($\Delta E_{00}$) [31] total color difference formulas as shown in Equations (1) and (2), respectively:

(1) $$\Delta E_{ab}^{*}=\sqrt{{\left(L_{1}^{*}-L_{2}^{*}\right)}^{2}+{\left(a_{1}^{*}-a_{2}^{*}\right)}^{2}+{\left(b_{1}^{*}-c_{2}^{*}\right)}^{2}}$$

(2) $$\Delta E_{00}={\left[{\left(\frac{\Delta L^{\prime }}{k_{L}S_{L}}\right)}^{2}+{\left(\frac{\Delta C^{\prime }}{k_{C}S_{C}}\right)}^{2}+{\left(\frac{\Delta H^{\prime }}{k_{H}S_{H}}\right)}^{2}+R_{T}\left(\frac{\Delta C^{\prime }}{k_{C}S_{C}}\right)\left(\frac{\Delta H^{\prime }}{k_{H}S_{H}}\right)\right]}^{\frac{1}{2}}$$

$\Delta E_{ab}^{*}$ and $\Delta E_{00}$ values were evaluated according to the corresponding 50:50% perceptibility (PT) and acceptability (AT) thresholds for color difference in dentistry described on literature [32,33] and recommended by the ISO/TR 28642:2016 [34] (PT: $\Delta E_{ab}^{*}=1.2$, $\Delta E_{00}=0.8$; AT: $\Delta E_{ab}^{*}=2.7$, $\Delta E_{00}=1.8$).

## 3. Results

Table 1 shows the mean CIE L*a*b* values of the three thirds considered of the target teeth measured in this study.

A variety of CIE-L*a*b* color coordinates values of dental structures have been reported in several studies [8,28,35] where other capturing technologies were used to analyze the color changes within a tooth and between teeth. As a summary and in order to compare their results to those obtained in the current study, some key-parameters are presented in Table 2.

**Figure 3:**
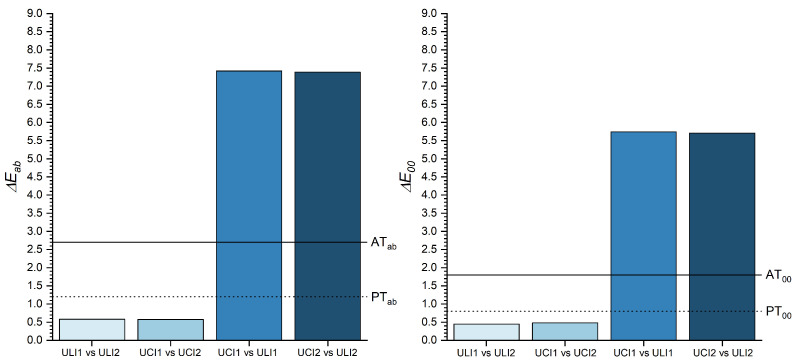
$\Delta E_{ab}^{*}$ and $\Delta E_{00}$ color differences between the contralateral and adjacent incisors.

In order to evaluate the differences within teeth of the same type -contralateral incisors- (UCI1-UCI2 and ULI1-ULI2) and those of adjacent incisors (UCI1-ULI1 and UCI2-ULI2), the overall $\Delta E_{ab}^{*}$ and $\Delta E_{00}$ color differences were calculated (Figure 3).

**Figure 4:**
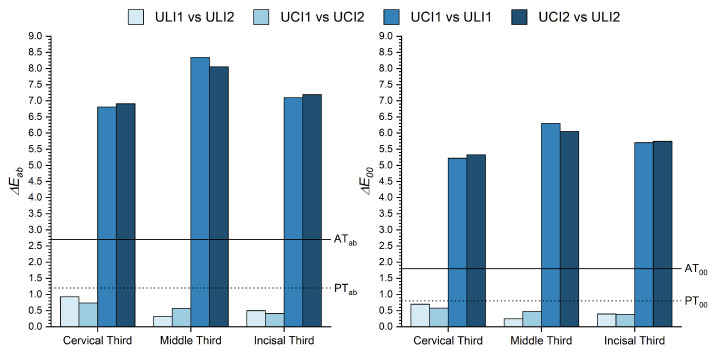
$\Delta E_{ab}^{*}$ and $\Delta E_{00}$ color differences between the cervical, middel and incisal thirds of contralateral and adjacent incisors.

Also, it was of interest to evaluate which of the three thirds presented higher differences. Figure 4 shows the $\Delta E_{ab}^{*}$ and $\Delta E_{00}$ color differences found between the same third of contralateral and adjacent teeth.

**Figure 5:**
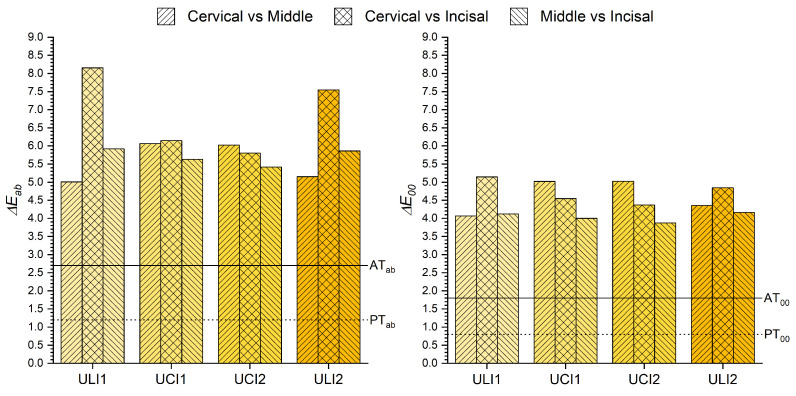
$\Delta E_{ab}^{*}$ and $\Delta E_{00}$ color differences between the cervical, middle and incisal thirds of the same tooth.

Finally, in order to assess the extent to which color differences between the three different tooth areas are perceived and/or accepted clinically, $\Delta E_{ab}^{*}$ and $\Delta E_{00}$ values were calculated between cervical and middle, middle and incisal and cervical and incisal tooth thirds. Results are shown in (Figure 5).

## 4. Discussion

Tooth color matching is a complex procedure and is considered one of the most difficult challenges in clinical dentistry. Different studies [8,9,10,11,28], demonstrated that tooth color is not uniform and that single teeth exhibit a variety of colors within its structure. Current devices have limitations while measuring these complex structures, and measuring the spectral reflectance data of complete tooth and evaluating these color changes in a precise way has not been fully investigated yet. Therefore, the study of the color variations between teeth and different regions of a tooth is still a matter of concern.

Different digital systems used in clinical scenarios can provide slightly different CIE-L*a*b* values, even if the same measurement conditions are reproduced [9,13,15]. Since the reflectance spectrum of any object is independent of illumination and viewing conditions, this is one of the best ways to describe its color. Therefore, in the last years, the research focus has shifted towards devices that measure reflectance rather than colorimetric values [20,23,24,36]. In this regard, hyperspectral imaging systems have the potential to provide whole surfaces spectral data and to the best of our knowledge, these have not been used before for objective color evaluation of in-vivo dental structures, therefore it is necessary to evaluate the performance of the proposed measuring system in order to ensure the reliability and accuracy of the proposed measuring protocol and data processing before more complex studies can be carried out. In the present study, a hyperspectral imaging system was used to capture the spectral reflectance of in-vivo maxillary anterior teeth in order to characterize the color correlations within contralateral and adjacent teeth and within the cervical, middle and incisal thirds and to compare the results to those previously described in literature.

Based on the results of this in-vivo study, the L*a*b* color coordinates values obtained are in agreement with those presented in other similar studies using different color measuring devices (Table 2). However, while for CIE-L* and CIE-b* coordinates the values are similar, for CIE-a* chromatic coordinate some slight differences were found [8,28,35]. In this regard, different studies attempted to establish a reference chromatic space for dentistry and determined that the limits for CIE-a* coordinates were a* = 1–6 [37] and a* = 1.7–8.9 [38] which means that the mean CIE-a* values obtained in our study (Table 1 and Table 2) fall within the defined limits of the dental color space.

Regarding the comparisons between the different dental structures analyzed with this capturing system, higher CIE-L* values were obtained for the two central incisors (UCI1 and UCI2) when compared to the corresponding lateral incisors (ULI1 and ULI2). Similarly, slightly higher values were obtained for the CIE-b* coordinate and lower values for the CIE-a* coordinate (Table 1). This means that, in chromatic terms, the central incisors have a brighter and less chromatic appearance than the lateral incisors. Also, when considering each of the three thirds individually, it is observed that, independently of the type of tooth analyzed, the CIE-L* coordinate (lightness) increases from the upper third to the middle third but then experiences a slight decrease in the lower third. Regarding the variations of the CIE-a* (red-green) and CIE-b* (yellow-blue) coordinates, these show a continuous decrease from the cervical third to the incisal third. This means that the cervical third of the natural central and lateral incisors has a reddish (higher CIE a* values) and more yellowish (higher CIE b* values) appearance than the middle and incisal thirds of the same tooth. This behavior can be explained not only by the proximity of the gingiva and the translucent nature of the natural tooth structures [36] (reddish appearance) but also by the dentine thickness in the cervical third which is thicker than in the middle and incisal ones (yellowish appearance). These results are in agreement with the results reported in other studies, where color correlations within a tooth and between different types of anterior teeth were studied [8,28,35]. However, the results obtained in these previous studies show slightly higher CIE-L* values than those found in the present study, for both central and lateral incisors. These variations may be due to the measurement geometry and, above all, to the instrument used for objective color measurement (hyperspectral camera versus clinically commercial devices). Moreover, it should be noted that the CIE-L*a*b* values found for the two central incisors and the two lateral incisors are very similar, again highlighting the chromatic symmetry that exists in the oral environment, as was also reported in [8].

This chromatic symmetry is also reflected in the values of the color differences found between the two central incisors ($\Delta E_{ab}^{*}=0.57$ and $\Delta E_{00}=0.48$) and the lateral incisors ($\Delta E_{ab}^{*}=0.58$ and $\Delta E_{00}=0.45$) as shown in Figure 3. In both comparisons, the color differences found are, regardless of the color difference formula used for their calculation, lower than the perceptibility threshold [32]. This means that the chromatic difference between the two central and the two lateral incisors, is imperceptible to an average observer. This finding is very helpful in clinical practice, since it implies that, in the case of performing a restoration of one of the four maxillary anterior teeth -specially if this is completely absent- the color of its respective contralateral tooth can be used as a reference.

Nonetheless, the color differences between adjacent teeth (central incisors versus lateral incisors), were considerably higher than those found between contralateral teeth ($\Delta E_{ab}^{*}=7.42$ and $\Delta E_{00}=5.74$ between UCI1-ULI1; and $\Delta E_{ab}^{*}=7.39$ and $\Delta E_{00}=5.71$ between UCI2-ULI2) as shown in Figure 3, which exceeded, in all cases, their corresponding acceptability thresholds (AT) established for dentistry. Color differences above the AT between central incisors and lateral incisors have been previously found in other studies [35].

Also, when comparing the differences found between the same third of different teeth, similar results are found (Figure 4). While, for contralateral teeth both $\Delta E_{ab}^{*}$ and $\Delta E_{00}$ are always below PT for the three thirds, the color differences found between UCI1-ULI1 and UCI2-ULI2 exceed greatly the corresponding AT. These color differences also varied according to the dental third being analyzed. While smaller differences were found for the cervical third ($\Delta E_{ab}^{*}$ = 6.81–6.91 and $\Delta E_{00}$ = 5.22–5.33), slightly higher differences were found for the incisal third ($\Delta E_{ab}^{*}$ = 7.10–7.19 and $\Delta E_{00}$ = 5.71–5.75) and considerably higher for the middle third ($\Delta E_{ab}^{*}$ = 8.06–8.34 and $\Delta E_{00}$ = 6.05–6.30), which, in fact, it is the one used as the reference for visual color matching. Therefore, the first research hypothesis is accepted, since the differences found between contralateral and adjacent teeth were below PT and above AT, respectively.

Lastly, the color differences between different thirds of the same tooth were also studied. Similarly to what was reported in other studies [8], in Figure 5 it can be observed that there are big differences between different areas of the same tooth, being these always above AT, independently of the color difference formula used. However, there are disparities between the results found for lateral and central teeth. While for central teeth the highest differences were found between cervical and middle thirds ($\Delta E_{ab}^{*}$ = 6.02–6.15 and $\Delta E_{00}$ = 5.02–5.03), for lateral teeth the highest differences were found between cervical and incisal thirds ($\Delta E_{ab}^{*}$ = 7.54–8.16 and $\Delta E_{00}$ = 4.84–7.54). These results are of significant clinical relevance, since they corroborate that there are large variations between different thirds of the same tooth, and also that there is a chromatic gradient along the cervical-incisal axis of the tooth [28]. Therefore, the second research hypothesis was accepted.

Hyperspectral imaging of dental structures allows acquisition of a large amount of data from a single shot. One of the main advantages of using a hyperspectal imaging in dentistry is its short capturing time, which overcomes the problem of tooth dehydration and related color changes [29]. In this sense, the proposed method has proven to be valid for color characterization in dentistry, since our results are in accordance with those obtained in other studies [8,28,35]. However, there will still be some limitations such as the difficulty of dealing with huge amounts of measured data and the specular reflections that may occur during the capturing process. These specular phenomena can also appear when other non-contact measuring devices are used, and, in this regard, due to the amount of data acquired, hyperspectral capturing systems have the advantage that areas not including specular reflections can be selected for data processing. Also, although successful tooth color reproduction can be achieved with the proposed method, it needs specific adaptation to be performed in a chairside scenario. Besides, novel devices including hyperspectral imaging measurements would need to be developed for it to be directly transferred to the clinical practice. Nonetheless, the findings of this study can be an educational aid for dentists, technicians and dental materials companies to achieve better color reproduction in dental restorations and in the fabrication of new materials and prosthetic teeth. In future studies, it would be interesting to evaluate the correlation between different areas of the same tooth in order to model and predict the existing chromatic gradient. This could help provide dentists with a guide to carry out dental restorations of a missing tooth area based on the color and appearance of another existing area. Also, it would be interesting to use this method in order to build a chromatic map of dental structures, so future studies could include a more comprehensive analysis, with more than three areas of given tooth ore more than the four upper incisors, to better understand chromatic gradient within a given tooth as well as color relationships between different teeth. In this sense, Artificial Intelligence (AI) and Deep Learning (DL) methods could definitely contribute to improve these hyperspectral measuring systems in the future, by integrating Artificial Neural Network learning procedures or Hierarchical Convolutional Neural Network into its measuring procedure, and mostly, into post-processing of measured data and data display [17,39] Additionally, future studies could include more patients, with a wider age range and with more representative age groups. Finally, teeth with chromatic disorders could also be included in order to better understand the colorimetric variations that occur in such situations.

## 5. Conclusions

Within the limitations of the present study, such as the increased complexity of the proposed measurement system as compared to the simpler ones currently used, we can conclude that the use of a hyperspectral camera has proven to be a reliable and effective method for measuring the spectral reflectance (and consequent color calculation) of in-vivo natural teeth, since the following conclusions were drawn:

1. The color differences between adjacent teeth exceed the acceptability threshold for adjacent teeth but are lower than the perceptibility threshold between contralateral teeth.
2. The color differences between the different parts (thirds) of the same tooth were always higher than the color difference acceptability threshold for dentistry.

Thus, it is important for dental specialists and companies to consider these variations, especially when performing partial restorations of teeth or developing new prosthetic teeth or dental restorative materials. Future work should include a deeper analysis of chromatic changes within dental structures and a method to determine the chromatic map of a complete tooth could be described for this capturing system.

## Figures and Tables

**Table 1:** CIE-L*a*b* mean values and standard deviations (SD) for each third of the 4 target teeth measured in this study.

	ULI1			UCI1			UCI2			ULI2		
	L*	a*	b*	L*	a*	b*	L*	a*	b*	L*	a*	b*
**Cervical**	68.78 (4.58)	8.60 (1.38)	19.36 (2.54)	75.34 (5.30)	6.84 (1.14)	18.85 (2.83)	74.70 (5.30)	7.08 (1.34)	18.58 (2.93)	67.97 (4.90)	8.61 (1.12)	18.90 (2.69)
**Middle**	70.95 (4.89)	4.79 (1.00)	16.94 (2.89)	79.02 (5.67)	2.82 (0.77)	16.18 (2.61)	78.51 (5.58)	3.08 (0.98)	16.18 (3.04)	70.65 (5.29)	4.71 (1.03)	16.86 (2.63)
**Incisal**	67.01 (4.65)	4.51 (1.07)	12.53 (2.66)	73.70 (4.64)	2.99 (1.07)	14.35 (2.75)	73.39 (5.08)	3.25 (1.16)	14.42 (2.90)	66.54 (5.48)	4.59 (1.47)	12.68 (2.68)

**Table 2:** Overview of means and standard deviations of Upper Central Incisors CIE-L*a*b* values obtained with different color measuring systems used for tooth color determinations in Dentistry.

Study	Color Measurement System	Sample Size	L*	a*	b*
Djozic et al. [28]	Digital photography	50	73.8 (5.7)	−1.3 (1.4)	15.9 (2.8)
Djozic et al. [8]	Digital photography	100	83 (*)	−6.00 (*)	18.00 (*)
Turgut et al. [35]	Colorimeter	640	80.5 (5.1)	−0.2 (0.4)	17.1 (3.1)
Current	Hypespectral Imaging	60	78.76 (5.62)	2.95 (0.87)	16.18 (2.83)

* Standard deviation values are not given in the study.

## Data Availability

Not applicable.
